# Dietary Behavior and Physical Activity in Children and Adolescents

**DOI:** 10.3390/nu11081849

**Published:** 2019-08-09

**Authors:** Antje Hebestreit, Leonie H. Bogl

**Affiliations:** 1 Leibniz Institute for Prevention Research and Epidemiology—BIPS, D-28359 Bremen, Germany; 2 Department of Epidemiology, Center for Public Health, Medical University of Vienna, Kinderspitalgasse 15, 1. Floor, A-1090 Vienna, Austria

**Keywords:** dietary behavior, physical activity, young populations, surveillance, epidemiology, public health

In recent years, diet- and lifestyle-related disorders have become a major health threat in Europe and worldwide. Globally, optimal infant and child feeding practices include exclusive breastfeeding for the first six months of life, age-appropriate and safe complementary feeding, and the prevention of micronutrient deficiencies; they also target a balanced diet during childhood and adolescence to prevent childhood obesity and associated cardiovascular and metabolic sequelae [1]. The promotion of as well as a general increase in physical activity across all ages is one target across numerous settings and communities [2]. Contributions in this monograph include two review articles [3,4] and 19 original contributions from several countries, providing new information to existing research and elucidating important aspects of children’s and adolescents’ nutrition and lifestyle behavior.

Data included in this Special Issue are from large epidemiological studies including several multi-center [5,6] and multinational studies [3,7] as well as datasets from surveillance initiatives, such as the German Health Interview and Examination Survey for Children and Adolescents (KiGGS) [8,9], the U.S. National Health and Nutrition Examination Survey (NHANES) [10], and the WHO European Childhood Obesity Surveillance Initiative (WHO COSI) [11]. Three of the studies in this Special Issue reported on the co-occurrence of multiple health behaviors in the same children [6,8,11], in particular the clustering of low levels of physical activity levels and/or high screen times with a higher consumption of energy-dense foods [6,8]. These findings encourage future community-based intervention studies to target multiple lifestyle behaviors simultaneously to reduce the burden of lifestyle-related diseases.

The role of parenting and early feeding practices was investigated in four studies within this Special Issue [5,12,13,14]. Of particular interest, Walton K. et al. videotaped families during dinner and reported associations between food parenting practices and preschooler’s risk of poor nutrition. Physical restriction of food from a child was associated with higher nutrition risk whereas positive comments about a child’s food was associated with a lower nutrition risk in children [12]. These results suggest that the use of positive encouragement by supporting parents, rather than restriction, may improve preschoolers’ nutrition; they further highlight the previously described role of parents as gatekeepers [15].

Ensuring validity of dietary intake data is a prerequisite for any investigation into diet–disease associations. Three U.S. studies [10,16,17] and one Finnish study [18] focused on the validity of self-reported dietary intake data and derived dietary patterns. Validity refers to the extent to which a measurement actually measures what it is intended to measure. There are several types of validity, including criterion-related validity, construct validity, content validity, and face validity [19]. Hibbs-Shipp S.K. et al. [16] derived a diet quality score for the home food environment and carefully evaluated all four validity measures. Face validity was evaluated by expert reviews of representative foods and food amounts, considering whether representative foods can be realistically found in the target population’s home. Content validity was assessed by iterative runs and via the removal of each food individually from the score database to determine whether the representative food was loading into the component scores as theorized Criterion validity was assessed by testing the contributions of each food to component and total scores. Finally, construct validity was evaluated by testing five hypothetical home food environments that resulted in a range of scores in the expected directions. Using a validated FFQ, Wolters M. et al. confirmed a positive association between frequent fast food consumption and adverse changes in body composition indicators in two German pediatric cohorts [20].

Two European studies evaluated the prevalence and determinants of dietary supplement use in children and adolescents, an important research question given that dietary supplement use has been implicated in preventable adverse drug events and emergency department visits in children and adolescents [21,22]. Data from the KiGGS Module EsKiMo II study showed that around 16% of adolescents in Germany use dietary supplements [9], and data from Eastern Poland reported that around 30% of children and adolescents use vitamin or mineral supplements [23]. Notably, the time frame for which supplement intake was queried was different in these two studies; in Germany the time frame was the previous four weeks and in Poland the past 12 months. In comparison, 33% of children and adolescents in the United States use dietary supplements [21]. In the two European studies, supplement users were more often female, living in urban areas, from more highly educated families, more likely to be physically active, and less likely to be overweight or obese [9,23].

Growing up in lower socio-economic environments or vulnerable groups such as ethnic minorities may increase susceptibility to unhealthy dietary patterns [24]. The study in this Special Issue from New Caledonia reported that the proportion of adolescents regularly consuming sugar-sweetened beverages was high (90%) and was related to living in rural areas and belonging to a particular ethnic community [25]. Results from the WHO Health Behavior in School-Aged Children survey in Belgium are in line with previous findings which showed that eating culture plays a role in inequalities of eating habits among immigrants when socioeconomic conditions are considered [26]. Mustafa N. et al. [27] further highlighted that the type of foods consumed at breakfast are highly dependent on culture. Malaysian adolescents consume breakfast that is of low nutrient quality, such as cereal-based and often primarily rice dishes, chocolate and confectionary, hot and powered drinks, and noodles. The Malaysian study suggests that breakfast consumption is related to lower cardiovascular risk because of the earlier timing of food intake rather than the types of foods consumed at breakfast. This interesting hypothesis remains to be established in randomized trials.

The diverse articles in this Special Issue highlight the complexity and extent to which nutrition and physical activity behaviors may influence different health aspects of children and adolescents. Few studies in this Special Issue combined genetic data with nutritional data [28], a likely expanding research area in the coming years, with the goal to provide personalized and gene-based dietary recommendations in the future. As seen by the various findings and recommendations, not only is more work in this area required but the translation of this work to practice and policy is imperative if we are to address the challenges impacting the nutrition, physical activity, and health of young populations.
